# Whitening-Aided Learning from Radar Micro-Doppler Signatures for Human Activity Recognition †

**DOI:** 10.3390/s23177486

**Published:** 2023-08-28

**Authors:** Zahra Sadeghi Adl, Fauzia Ahmad

**Affiliations:** Department of Electrical and Computer Engineering, Temple University, Philadelphia, PA 19122, USA; zahra.sadeghi.adl@temple.edu

**Keywords:** whitening, convolutional neural network, human activity recognition, micro-Doppler, deep learning

## Abstract

Deep learning architectures are being increasingly adopted for human activity recognition using radar technology. A majority of these architectures are based on convolutional neural networks (CNNs) and accept radar micro-Doppler signatures as input. The state-of-the-art CNN-based models employ batch normalization (BN) to optimize network training and improve generalization. In this paper, we present whitening-aided CNN models for classifying human activities with radar sensors. We replace BN layers in a CNN model with whitening layers, which is shown to improve the model’s accuracy by not only centering and scaling activations, similar to BN, but also decorrelating them. We also exploit the rotational freedom afforded by whitening matrices to align the whitened activations in the latent space with the corresponding activity classes. Using real data measurements of six different activities, we show that whitening provides superior performance over BN in terms of classification accuracy for a CNN-based classifier. This demonstrates the potential of whitening-aided CNN models to provide enhanced human activity recognition with radar sensors.

## 1. Introduction

Owing to its privacy-aware nature and robustness against a variety of operating conditions, radar technology is finding increasing applications in healthcare [1,2,3,4,5,6,7,8,9,10,11]. These include remote patient monitoring outside of a hospital setting, rehabilitation interventions with a focus on improving mobility, and eldercare for aging-in-place. From an algorithmic perspective, human activity recognition is a core characteristic of radar-sensing solutions for such applications.

Classification of human activities using radar has recently experienced an influx of deep learning models due to their predictive power and ability to automatically learn relevant discriminant features from radar measurements [12,13,14,15,16,17,18]. In particular, convolutional neural networks (CNNs) are being extensively used for learning spatial hierarchies of features from micro-Doppler signatures of human activities [19,20,21,22,23,24,25,26]. In [19], a four-layer CNN-based activity classifier was used with Cepstral heatmaps, which were computed from the real radar spectrograms by applying an optimized filter bank generated on a diversified simulation database. A flexible deep CNN model was proposed in [20] to classify Doppler signatures of humans walking with different arm movements. Therein, a Bayesian learning technique was used to optimize the network. In [21], a dot-product attention-augmented convolutional autoencoder was proposed to learn both localized information and global features from micro-Doppler signatures. Superior classification accuracy was achieved by the attention-augmented model compared to its conventional counterpart. In [22], AlexNet was trained with an attention module to learn to highlight salient regions in micro-Doppler signatures, which in turn was shown to enhance the network predictions. A hybrid model comprising a long short-term memory (LSTM) network and a one-dimensional CNN, was introduced in [23], which provided enhanced classifications of human activities with relatively low complexity over two-dimensional (2-D) CNN methods. Complex-valued CNN-based architectures were investigated in [24] with micro-Doppler signatures, range–time plots, and range–Doppler maps as the data formats of choice. Using experimental data of nine human activities, the advantages of complex-valued models over their real-valued counterparts were demonstrated for certain data formats and network architectures. In [25], a multi-view CNN and LSTM hybrid network was proposed for human activity recognition, which fused multiple views of the time-range-Doppler radar data-cube. In [26], a millimeter-wave radar was used for real-time contactless fitness tracking via deep CNNs, providing an effective alternative to body wearable fitness trackers.

Most CNN-based solutions for recognizing human activities with radar readily employ batch normalization (BN) [27], which standardizes the activations of each batch in a layer. This renders the loss function considerably smooth, which in turn leads to improved accuracy and training speed for gradient-based methods [28]. Benefits beyond those afforded by BN in terms of model optimization and generalization can be achieved by whitening the hidden layers’ activations [29]. However, to the best of our knowledge, the impact of decorrelating the activations by whitening has not been investigated for the application at hand. In this paper, we propose the use of a whitening-aided CNN to effectively distinguish between radar micro-Doppler signatures of different human activities. We employ the iterative batch normalization (IterNorm) technique [30] which uses Newton’s iterations to efficiently implement whitening, thereby avoiding the high computational load imposed by eigen-decomposition of the data covariance matrix required otherwise. Convergence of IterNorm is guaranteed by normalizing the eigenvalues of the covariance matrix. Additionally, following the work in [31], we exploit the rotational freedom afforded by the whitening matrix to design an add-on rotation module, which can align different activity classes in orthogonal directions in the latent space. We test two different whitening-aided CNN models, one exploiting IterNorm only in lieu of BN layers and the other replacing BN layers with IterNorm + rotation module, on real data measurements of six different activities, namely, sitting down, standing up, walking, drinking water, bending to pick up an object, and falling. We show that whitening the latent space of a model provides significant enhancements to the classification accuracy compared to the CNN architecture with BN layers, with the alignment of the axes along the classes via rotation providing a slight advantage over the IterNorm only model.

The remainder of the paper is organized as follows. Section 2 describes the radar signal model and the micro-Doppler signatures. The BN and whitening methods are presented in Section 3, while the whitening-aided CNN models for human activity classification are described in Section 4. With the aid of real data examples, we demonstrate in Section 5 the usefulness of the whitening-aided models in achieving higher classification accuracy and also provide insights into the achieved performance enhancements over a base model employing BN layers. Concluding remarks are provided in Section 6.

## 2. Signal Model and Micro-Doppler Signatures

Consider a frequency-modulated continuous-wave (FMCW) radar, with the transmit signal, $s_{T}\left(t\right)$, given by

(1) $$s_{T}\left(t\right)=A_{T}\left(t\right)\cos\left[2\pi \left(f_{c}t+\frac{1}{2}\alpha t^{2}\right)\right],$$

where $A_{T}\left(t\right)$ is the signal amplitude, $f_{c}$ is the carrier frequency, and α is the chirp rate. For a moving point target, the radar return, $s_{R}\left(t\right)$, can be expressed as

(2) $$s_{R}\left(t\right)=A_{R}\left(t\right)\cos\left[2\pi \left(f_{c}\left(t-\tau \right)+\alpha \left(\frac{1}{2}t^{2}-\tau t\right)+f_{D}t\right)\right],$$

where $A_{R}\left(t\right)$ is the received signal amplitude, τ is the two-way travel time, and $f_{D}$ is the Doppler shift. The in-phase (*I*) and quadrature-phase (*Q*) components of the complex baseband signal can be obtained by demodulating $s_{R}\left(t\right)$ using the *I*/*Q* demodulator as

(3) $$s\left(t\right)=I\left(t\right)+jQ\left(t\right)=A\left(t\right)e^{j2\pi (\left(f_{D}-\alpha \tau \right)t-f_{c}\tau )},$$

where A(t) is the amplitude of s(t).

For the activity recognition problem, the human body can be viewed as a collection of moving point scatterers, which results in the corresponding radar return being a superposition of individual returns of the form of (3), represented by

(4) $$s\left(t\right)=\sum_{i}A_{i}\left(t\right)e^{j2\pi (\left(f_{D_{i}}-\alpha \tau_{i}\right)t-f_{c}\tau_{i})},$$

where $A_{i}\left(t\right)$ is the amplitude, $f_{D_{i}}$ is the Doppler frequency, and $\tau_{i}$ is the two-way travel time, all corresponding to the *i*th point scatterer.

Once the complex baseband signal has been sampled, it can be arranged as a 2-D matrix, $s(n_{1},n_{2})$, with $n_{1}$ and $n_{2}$ denoting fast-time and slow-time, respectively. To compute the range map, $R(p,n_{2})$, we take the discrete Fourier transform (DFT) along the matrix columns, represented by

(5) $$R\left(p,n_{2}\right)=\frac{1}{N_{1}}\sum_{n_{1}=0}^{N_{1}-1}s\left(n_{1},n_{2}\right)e^{-j(2\pi pn_{1}/N_{1})},$$

where $N_{1}$ is the number of samples (range bins) in one pulse repetition interval, p=0,$1,\ldots ,N_{1}-1$, and $n_{2}=0,1,\ldots ,N_{2}-1$, with $N_{2}$ representing the total number of considered pulse repetition intervals. Next, the corresponding micro-Doppler signature is obtained through a two-step process. First, we sum the data over the range bins of interest as

(6) $$v\left(n_{2}\right)=\sum_{p=p_{1}}^{p_{2}}R\left(p,n_{2}\right),$$

with $p_{1}$ and $p_{2}$ being the minimum and maximum range bins considered. Then, we apply the Short-Time Fourier Transform (STFT) to $v(n_{2})$ and compute the micro-Doppler signature, $D(k_{1},k_{2})$, as the spectrogram (the squared-magnitude of the STFT). That is,

(7) $$D\left(k_{1},k_{2}\right)={\left|\sum_{n=0}^{N-1}v\left(n+k_{1}h\right)w\left(n\right)e^{-j(2\pi nk_{2}/N)}\right|}^{2},$$

where w(n) represents the window of length $N(<N_{2})$ that determines the trade-off between time and frequency resolutions [32], the integer *h* determines the step size by which the window is shifted across the signal $v(n_{2})$, $k_{1}$ is the time index. and $k_{2}$ is the frequency index. These micro-Doppler signatures serve as the input to the CNN-based classifier for human activity recognition.

## 3. Whitening Methods

We briefly review BN and present two whitening methods, which form integral algorithmic components of the proposed whitening-aided CNN-based models for classification of human activities.

### 3.1. Batch Normalization

Let $X\in R^{d\times m}$ be the batch input of a layer, with *d* denoting the dimension of the layer’s vector input and *m* representing the number of samples in the batch. BN operation first centers and scales X to produce a standardized output $X_{S}$ as

(8) $$\begin{array}{cc} X_{S} & =\Lambda_{s}^{-\frac{1}{2}}X_{C}, \end{array}$$

(9) $$\begin{array}{cc} X_{C} & =X-\mu \cdot 1_{m}^{T}, \end{array}$$

where the matrix $\Lambda_{s}=\mathrm{diag}\left(\sigma_{1}^{2},...,\sigma_{d}^{2}\right)+\epsilon I_{d}$ contains the batch variances $\sigma_{i}^{2}$ corresponding to the *i*th input dimension and incorporates diagonal loading for numerical stability via the second term, $I_{d}$ is an identity matrix of size *d*, ϵ>0 is the diagonal loading factor, $\mu \in R^{d}$ is the batch mean given by

(10) $$\mu =\frac{1}{m}X\cdot 1_{m},$$

$1_{m}$ is an m×1 column vector of all ones, and the superscript ${\left(\cdot \right)}^{T}$ denotes matrix transpose. Each column of $X_{S}$ has zero mean and unit variance for each dimension. To ensure that BN represents an identity transformation when inserted in the deep learning model, a scale parameter $\alpha \in R^{d}$ and a shift parameter $\beta \in R^{d}$ are introduced to yield the output of the BN layer as [27]

(11) $$X_{BN}=\alpha \cdot 1_{m}^{T}⊙X_{S}+\beta \cdot 1_{m}^{T},$$

where ‘⊙’ denotes the Hadamard product. Both α and β are learned during model training to restore its representation power; see [27] for more details.

### 3.2. Whitening Method 1: IterNorm Batch Whitening

The output of a whitening layer is obtained by centering and decorrelating the batch input X through a d×d whitening matrix W as

(12) $$X_{W}=WX_{C},\;\;W^{T}W=\Sigma^{-1}$$

where Σ is the covariance matrix of X, $X_{C}$ is defined in (9), and ${\left(\cdot \right)}^{-1}$ denotes the matrix inverse. The constraint in (12), however, does not uniquely determine W [33]. A popular choice for the whitening matrix is given by

(13) $$W=\Sigma^{-1/2},$$

where ${\left(\cdot \right)}^{-1/2}$ denotes the inverse square-root of the matrix argument. Typically, the eigen-decomposition of Σ is used to determine $\Sigma^{-1/2}$. However, the eigen-decomposition is computationally demanding and can excessively increase the computational complexity of the deep learning model. Instead, the efficient IterNorm batch whitening [30] can be employed which uses Newton’s method to iteratively compute the whitening matrix W.

The IterNorm technique is provided in Algorithm 1. The batch mean μ is computed using (10) in line 1, followed by the centered activations $X_{C}$ using (9) in line 2. The covariance matrix Σ is estimated in line 3 as $\frac{1}{m}X_{C}X_{C}^{T}+\epsilon I_{d}$, where the second term represents diagonal loading for numerical stability. Next, in lines 5 through 8, the algorithm estimates the whitening matrix iteratively using

(14) $$\begin{array}{ccc} P_{0} & = & I_{d},\\ P_{k} & = & \frac{1}{2}\left(3P_{k-1}-P_{k-1}^{3}\Sigma \right),\;k=1,2,\ldots ,K, \end{array}$$

where $P_{k}$ is the estimated whitening matrix at the *k*th iteration and *K* is the total number of iterations. We note that to guarantee convergence under a limited batch size, IterNorm uses trace-normalized covariance matrix, $\Sigma_{N}$, instead of Σ, in (14). This is evident from line 7 of Algorithm 1, with $\Sigma_{N}$ calculated in line 4 as Σ/tr(Σ), where tr(·) denotes the trace of its matrix argument. At the end of *K* iterations, the whitening matrix W is calculated using $P_{K}$ in line 9, which is finally utilized together with $X_{C}$ to compute the whitened output, $X_{W}$, in line 10.
**Algorithm 1** IterNorm Batch Whitening Algorithm [30].     **Input**: Batch input $X\in R^{d\times m}$     **Hyperparameters**: constant ϵ and number of iterations *K*     **Output**: Whitened activations $X_{W}$ 1:Calculate batch mean μ using (10) 2:Calculate centered activations $X_{C}$ using (9) 3:Calculate the covariance matrix Σ as $\frac{1}{m}X_{C}X_{C}^{T}+\epsilon I_{d}$ 4:Calculate the trace-normalized covariance matrix $\Sigma_{N}=\Sigma /\mathrm{tr}\left(\Sigma \right)$ 5:$P_{0}=I_{d}$ 6:**for** k=1 to *K* **do** 7:    $P_{k}=\frac{1}{2}\left({3P}_{k-1}-P_{k-1}^{3}\Sigma_{N}\right)$ 8:**end for** 9:Calculate the whitening matrix: $W=P_{K}/\sqrt{\mathrm{tr}(\Sigma )}$10:Calculate whitened output: $X_{W}=WX_{C}$

### 3.3. Whitening Method 2: IterNorm + Rotation

While the constraint in (12) does not uniquely specify the whitening matrix W, it does enable rotational freedom. That is,

(15) $$\tilde{W}=Q^{T}W,$$

is also a valid whitening matrix, provided Q is an orthogonal matrix with $Q^{T}Q=I_{d}$. Equation (15) interprets whitening as a combination of rescaling by W of (13) and rotation by Q [33].

Following the work in [31] and assuming that we are interested in recognizing $N_{c}$ classes of human activity, we align the *i*th column $q_{i}$ of the orthogonal matrix Q with class $c_{i}$. That is, after whitening the activations using W obtained via IterNorm, we employ Q to rotate the samples such that the data corresponding to class $c_{i}$ is maximally activated along $q_{i}$. Such a matrix Q can be determined by solving the optimization problem [31]

(16) $$\begin{array}{c} \max_{q_{1},q_{2},...q_{N_{c}}}\sum_{i=1}^{N_{c}}\frac{1}{m_{i}}q_{i}^{T}X_{W,c_{i}}1_{m_{i}},\\ \mathrm{subject}\;\mathrm{to}\;Q^{T}Q=I_{d} \end{array}$$

where $X_{W,c_{i}}\in R^{d\times m_{i}}$ denotes the activations corresponding to class $c_{i}$ after whitening with W and $m_{i}$ is the number of samples for class $c_{i}$. The problem in (16) with orthogonality constraint can be solved via gradient-based approaches on the Stiefel manifold [31,34].

## 4. Whitening-Aided CNN-Based Activity Classification

Having described the whitening methods, we are now in a position to present the whitening-aided CNN-models for human activity recognition.

**Figure 1:**
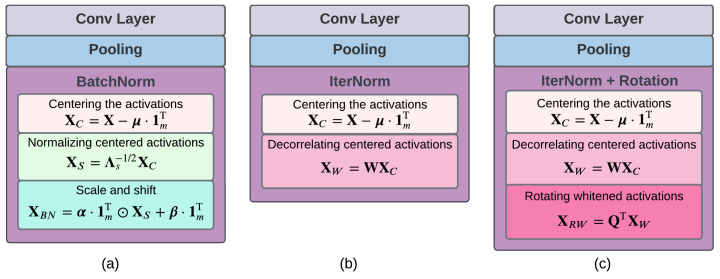
Building blocks of the various CNN models, differing in terms of the employed normalization layer. (**a**) Base CNN model employing BatchNorm layer to center and normalize the input. (**b**) Whitening-aided model 1 using IterNorm whitening module to decorrelate the centered input. (**c**) Whitening-aided model 2 which adds a rotation module after the IterNorm module to maximize the class activations along the axes of the whitened latent space.

We consider a base CNN model consisting of a series of building blocks. Each building block comprises a convolutional layer, followed by a max-pooling layer and then a BN layer, as seen in Figure 1a. Each convolutional layer generates feature maps by convolving its input with 2-D filters in a sliding window fashion and then feeding the filter outputs to an activation function. Considering a convolutional layer with *L* filters and denoting the input of the convolutional layer by $C\in R^{h_{t}\times w_{t}}$, we can express the *l*th convolutional map, $O^{\left(l\right)}\in R^{h_{c}\times w_{c}}$, corresponding to the *l*th filter as

(17) $$O^{\left(l\right)}=\sigma \left(C*f^{\left(l\right)}+b^{\left(l\right)}\right),$$

where ‘∗’ denotes 2-D convolution, σ is the activation function, $b^{\left(l\right)}$ is the bias term corresponding to the *l*th map, and $f^{\left(l\right)}\in R^{h_{f}\times w_{f}}$ is the *l*th 2-D convolutional filter. Next, the max-pooling layer downsamples the feature maps by taking the maximum over an $h_{p}\times w_{p}$ spatial window for complexity reduction [35]. Finally, the BN layer applies centering and scaling operations to normalize the downsampled feature maps within a batch. We note that the micro-Doppler signature of (7) serves as the input of the first building block, whereas the input of each subsequent block is the output of the previous block.

A whitening-aided CNN model is essentially the same as the base CNN model with the exception that it employs a whitening layer in lieu of BN in its building blocks. We consider two whitening-aided models, namely, whitening-aided models 1 and 2; the former replaces BN layer with an IterNorm layer as shown in Figure 1b, whereas the latter employs IterNorm + Rotation in place of BN as depicted in Figure 1c.

We note that in Section 3, the activations for the BN and whitening methods are assumed to be vectors. However, the output of a convolutional layer comprises a total of *L* 2-D feature maps. As such, the batch input to any normalization layer in this case would be of size $h_{d}\times w_{d}\times L\times m$, where $h_{d}$ and $w_{d}$ indicate the height and width of the downsampled feature maps (output of the max-pooling layer) and *m* is the number of samples in the batch. Following [27,30,31], we unroll the batch input as $X\in L\times (mh_{d}w_{d})$. The BN and whitening operations can now proceed with the unrolled X as the batch input.

## 5. Experimental Results

In this section, we evaluate the performance of the whitening-aided CNN models for human activity classification using real data measurements. We compare the classification accuracy of the whitening-aided models with that of the base CNN model.

### 5.1. Experimental Dataset

**Figure 2:**
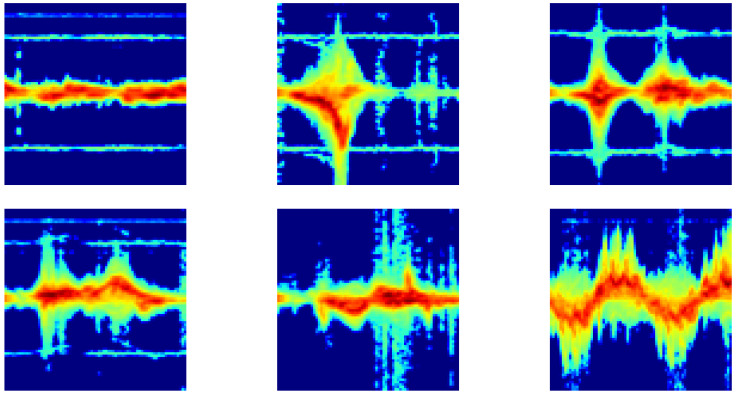
Micro-Doppler signatures of six human activities. Top row (from **left** to **right**): Drinking water, falling, and bending to pick up an object. Bottom row (from **left** to **right**): Sitting down, standing up, and walking. The horizontal axis denotes time whereas the vertical axis denotes Doppler frequency.

We employ the human activity dataset collected at the University of Glasgow, UK [36]. This dataset consists of six smaller subsets, out of which we employ the three subsets collected in 2017 in a laboratory environment. The data were collected using an FMCW radar, model SDR-KIT-580B by Ancortek (Fairfax, VA, USA), with a 5.8 GHz carrier frequency, 400 MHz bandwidth, and a chirp duration of 1 ms, delivering an output power of approximately 18 dBm. Two Yagi antennas, each with a gain of about 17 dB, were used for signal transmission and reception. The number of samples per recorded beat-note signal was set as 128. The dataset contains six activity classes: walking, sitting down, standing up, bending to pick up an object, drinking water, and falling. A total of 33 participants were used as test subjects, 31 of them were male and two were female, ranging in height from 149 cm to 188 cm with ages between 22 and 36 years. Each participant repeated each activity two to three times along the radar’s line of sight, i.e., measurements were made at normal incidence. The spectrograms were computed using a Hanning window length of 256 with 2048 frequency points and 254 points overlap, i.e., h=2 in (7). The resulting micro-Doppler signatures were then cropped, downscaled, and converted to grayscale images with dimensions of 75×75 and pixel values ranging from 0 to 255. The dataset contains a total of 570 micro-Doppler signatures, with 95 signatures per class. Representative signatures of each of the six activities are shown in Figure 2; the horizontal axis represents time while the vertical axis is Doppler frequency.

### 5.2. CNN Models and Training

**Figure 3:**
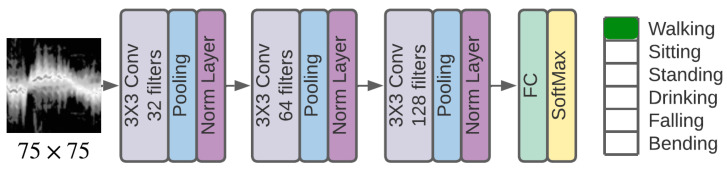
The 3-layer CNN model.

For illustration, we employ the learning architecture depicted in Figure 3, where the input to the network is a micro-Doppler signature of size 75×75. The network output is a one-hot encoded length-6 vector such that the location of a ‘1’ indicates a specific human activity. The input is passed through a 3-layer CNN implementing 32, 64, and 128 filters, respectively, each of kernel size 3×3. A max-pooling layer with a stride of 3 follows each convolutional layer. A normalization layer is the last module in each building block. A dropout layer (not shown in Figure 3) with a 15% rate is also included before the fully-connected output layer. The ReLU activation function is used for all layers except the output layer, which uses a softmax function. Three different variants of this learning architecture are considered, differing in terms of the employed normalization method, as detailed in Figure 1. Specifically, these include the base model with BN layers, whitening-aided model 1 with IterNorm layers, and whitening-aided model 2 with IterNorm + Rotation layers.

We utilize cross-entropy as the loss function for activity classification. To optimize the model, we apply stochastic gradient descent with a batch size of 10. We used an adaptive learning rate with an appropriate initial value for each CNN model, decreased by a factor of 10 after every seven epochs. A maximum of 30 epochs are used for training the base model and whitening-aided model 1, with the number of iterations for IterNorm set to 5. For whitening-aided model 2, we perform a warm start with the pretrained whitening-aided model 1 to which we add the rotation modules and continue the training for five additional epochs.

### 5.3. Classification Accuracy

**Figure 4:**
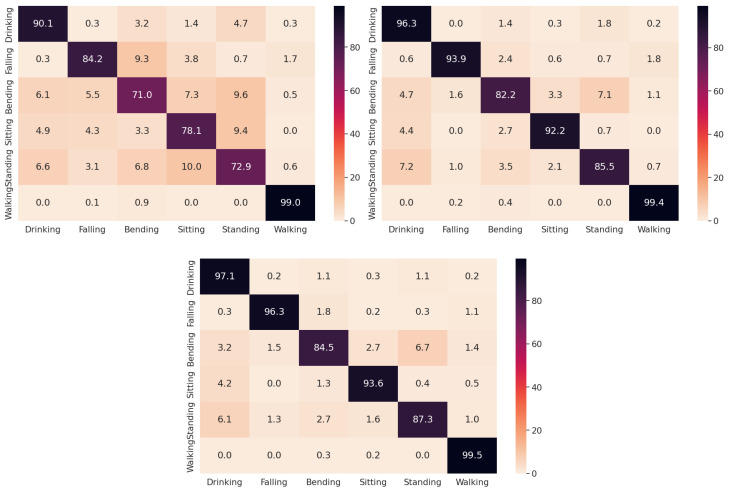
Confusion matrices of the base model (**top left**), whitening-aided model 1 (**top right**), and whitening-aided model 2 (**bottom**), for 50%/50% training/testing data split. Values are expressed in percentages.

We first examine the classification accuracy of the proposed whitening-aided models as a function of the number of training samples per class. We let the number of training samples vary from 20% to 80% in increments of 30%. The remaining signatures in each instance are utilized for testing. We conduct 30 classification experiments over distinct training and testing datasets for each considered split using the base CNN model and its whitening-aided counterparts. We calculate the mean and standard deviation of the test data classification accuracy for all three classifiers. The results are provided in Table 1. We clearly observe that for each training/testing split, both whitening-aided models significantly outperform the base model, especially under limited training samples. This is attributed to the reduced model confusion amongst the six classes resulting from the whitening of the latent space. The addition of the rotation module in whitening-aided model 2 to maximize the class activations along the latent space axes provides an additional 1.5% to 2% increase in average accuracy and relatively lower standard deviation values over whitening-aided model 1. This attests to further class disentanglement brought about by constraining the latent space to represent the classes. For further illustration of the impact of whitening, we compute the confusion matrices, averaged over 30 trials, corresponding to the base and the whitening-aided models for the 50%-50% training/testing data split. These confusion matrices, depicted in Figure 4, clearly demonstrate that the addition of the whitening layers cause a reduction in the model confusion for all six classes, with whitening-aided model 2 providing slightly higher reductions as compared to whitening-aided model 1.

Next, we consider 50%-50% training/testing data split and investigate the impact of whitening on the classification performance when introduced as a replacement for a single BN layer in the base model, leaving the remaining two BN layers intact. The corresponding average value and standard deviation of the classification accuracy are provided in Table 2, with the values corresponding to the base model under column labeled as “Base Model” and those corresponding to whitening methods 1 and 2 replacing BN in the first, second, and third layers of the network in respective columns labeled as “Layer 1”, “Layer 2”, and “Layer 3”. We observe that, compared to the base model, even replacing one BN layer with either whitening module yields performance enhancements, with progressively higher improvements for the introduction of the whitening layer at increasing depth of the network. Again, whitening method 2 provides higher accuracy on average and lower standard deviation as compared to whitening method 1. Comparing the results in Table 1 for 50%-50% training/testing data split and Table 2, we see that while replacing all BN layers with whitening layers yields the best performance, there is considerable value in replacing even a single BN layer with a whitening layer, especially deeper in the network and more so for whitening method 2 than method 1.

### 5.4. Correlation Coefficients

**Figure 5:**
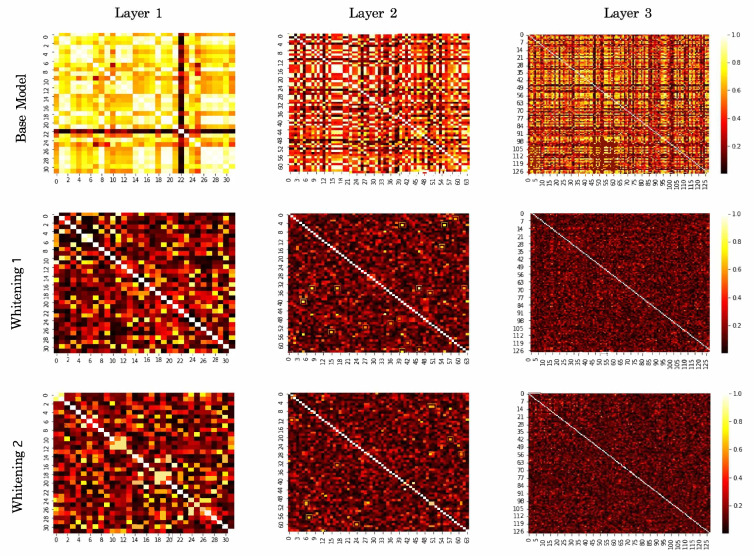
Absolute value of the correlation coefficient of every feature pair in the first, second and third layer for the base CNN model (**top row**), whitening-aided model 1 (**middle row**), and whitening-aided model 2 (**bottom row**).

To visually highlight the decorrelation aspect of the whitening layers, we consider the 50%-50% training/testing data split and measure the output of the normalization modules for the test set in each layer in the base model, whitening-aided model 1, and whitening-aided model 2 after training. We then calculate the absolute value of the correlation coefficient of every feature pair in each layer of the respective models. As depicted in the top row of Figure 5, the base model with all BN layers exhibits relatively strong correlations. This is expected since BN only standardizes the activations and does not decorrelate them. On the other hand, when all BN layers are replaced by either IterNorm layers or IterNorm+Rotation layers, the features in every layer indeed become decorrelated as seen in the middle and bottom rows of Figure 5, thereby leading to improved classification performance.

### 5.5. Top Activated Signatures

**Figure 6:**
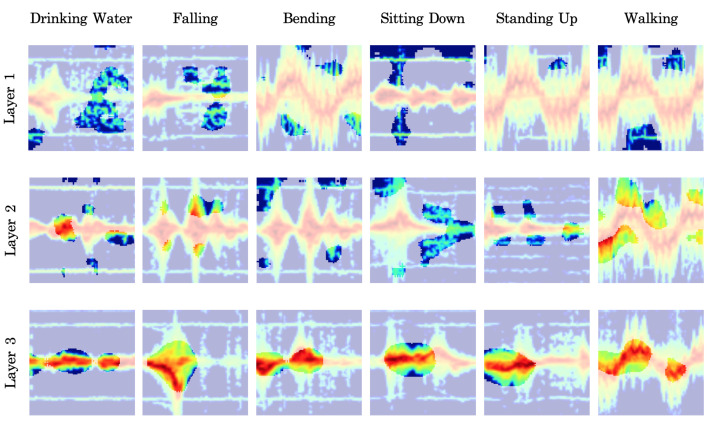
Most activated signatures in each axis in different layers for a trained Whitening-aided model 2 under 50%/50% training/testing data split. For each image, the empirical receptive field is also highlighted.

An important characteristic of whitening method 2 is its alignment of the axes of the latent space with the activity classes, which has been shown to enable an understanding of the learning process across the layers [31]. To this end, in this example, we assess the relationship between the test samples and a class label in the latent space for a trained whitening-aided model 2 with 50%-50% training/testing data split. We calculate the activation values of the test samples on each axis for each label and identify the top activated signature for each class in each layer, depicted in Figure 6. We observe that in the third layer, the top activated signatures correspond to the correct class labels. However, in the first layer, as the convolutional layers capture low-level information, the alignment is not as accurate as the higher levels. We also determine the empirical receptive fields of the top activated signatures by identifying those locations in each signature which when masked cause the largest reduction in the activation values on different latent space axes [31]. For this purpose, we apply 32×32 random masking patches with a stride of 5 on the top activated images. The corresponding results are shown as highlighted regions in Figure 6. Clearly, in the first layer, the extracted features appear to be related to the background, while by the third layer, the learned features are predominantly from the main pattern of the micro-Doppler signature. For example, the “Walking” axis in the third layer focuses on sinusoidal segments of the signature, while the “Falling” axis converges on the waterfall shape of the corresponding micro-Doppler signature.

### 5.6. Performance with Unseen Testing Data

In this final example, we examine the performance of the whitening-aided models under unseen testing data. Specifically, we retrain the networks using micro-Doppler signatures of 27 out of 33 human subjects (77 samples per class). The signatures of the remaining six subjects (18 samples per class), which were excluded from the training data, are used for testing. This is roughly equivalent to an 80%/20% training/testing data split. The respective classification accuracy values of the base model, whitening-aided model 1, and whitening-aided model 2 are 85.18%, 89.81%, and 92.59%. We note that the accuracy of each model is relatively lower than the corresponding average values reported in Table 2 for the 80%/20% data split. However, even in this case of unseen data, the superiority of the whitening-aided models over the base model is clearly evident, with whitening-aided model 2 outperforming whitening-aided model 1 as in the previous examples.

### 5.7. Summary of Findings

The above examples clearly demonstrate the superior performance of the whitening-aided CNN models over the base CNN model for human activity classification. The performance enhancements exist irrespective of testing with unseen data or samples from subjects the models have seen before during training. This superiority is attributed to the ability of the whitening layers to not only standardize but, more importantly, decorrelate the activations, and in case of whitening method 2 also to the alignment of the latent space axes with the activity classes. Further, while the results suggest replacing all BN layers in a CNN model with whitening layers to exploit their offerings to the fullest, considerable performance enhancements over the base model can be realized by using a whitening layer in lieu of even a single BN layer; the level of improvement increasing with increasing depth at which this replacement occurs in the network. Furthermore, performance evaluation of the two whitening methods showed that addition of the specific rotation module to IterNorm which maximizes the activation of the classes along the latent space axes provides model 2 with an appreciable advantage over model 1 in terms of classification accuracy, albeit at the additional expense of implementing the rotation module.

## 6. Conclusions

In this paper, we have presented CNN-based learning models that utilize whitening of the hidden layers’ activations for enhanced human activity recognition using radar. We employed IterNorm technique based on Newton’s method to significantly reduce the computational burden associated with the traditional eigen-decomposition approach for computing the whitening matrix. A rotation of the whitened activations to align the latent space axes with the corresponding class labels was also utilized. Results using real radar measurements of six different human activities were provided which validated the superior performance of the whitening-aided CNN models over the base CNN model in terms of classification accuracy. We also showed that the introduction of the specific rotation module can lead to appreciable improvements in classification accuracy over the IterNorm only layer. These findings demonstrate the potential of whitening-aided CNN models in enhancing the accuracy of human activity recognition using radar micro-Doppler signatures.

## Figures and Tables

**Table 1:** Classification accuracy of the 3-layer CNN model with and without whitening in all layers for different training/testing splits.

Split	Accuracy	Base Model	Whitening-Aided Model 1	Whitening-Aided Model 2
	Avg.	78.98	86.22	87.67
20/80	Std. Dev.	0.0412	0.0157	0.0121
	Avg.	82.54	91.59	93.05
50/50	Std. Dev.	0.0412	0.0163	0.0127
	Avg.	89.76	93.34	95.21
80/20	Std. Dev.	0.0401	0.0171	0.0132

**Table 2:** Classification accuracy of the 3-layer CNN model with and without Whitening.

		Whitening-Aided Models					
**Accuracy**	**Base Model**	**Layer 1**		**Layer 2**		**Layer 3**	
		**Method 1**	**Method 2**	**Method 1**	**Method 2**	**Method 1**	**Method 2**
Average	82.54	85.30	85.39	86.66	87.21	88.72	90.42
Std Dev.	0.0412	0.0253	0.0248	0.0238	0.0201	0.0198	0.0016

## Data Availability

Publicly available datasets from University of Glasgow, U.K. were used in this work. These datasets can be accessed at: http://researchdata.gla.ac.uk/id/eprint/848. Last accessed: 24 August 2023.

## Abbreviations

The following abbreviations are used in this manuscript:

2-D	Two-dimensional
BN	Batch Normalization
CNN	Convolutional Neural Network
DFT	Discrete Fourier Transform
FMCW	Frequency-modulated Continuous-wave
I/Q	In-phase/Quadrature-phase
IterNorm	Iterative Normalization
STFT	Short-time Fourier Transform
